# Sensing Characteristics of Tilted Long Period Fiber Gratings Inscribed by Infrared Femtosecond Laser

**DOI:** 10.3390/s18093003

**Published:** 2018-09-07

**Authors:** Jian Tang, Cailing Fu, Zhiyong Bai, Changrui Liao, Yiping Wang

**Affiliations:** 1 Key Laboratory of Optoelectronic Devices and Systems of Ministry of Education and Guangdong Province, College of Optoelectronic Engineering, Shenzhen University, Shenzhen 518060, China; tangjian2@email.szu.edu.cn (J.T.); fucailing89@163.com (C.F.); baizhiyong@szu.edu.cn (Z.B.); cliao@szu.edu.cn (C.L.); 2 Guangxi Colleges and Universities Key Laboratory of Microwave and Optical Wave-applied Technology, Guilin 541004, China

**Keywords:** femtosecond laser, TLPFG, optical fiber sensor, sensing properties

## Abstract

We propose a novel tilted long period fiber grating (TLPFG) design, inscribed using a line-by-line inscription technique and an infrared femtosecond (Fs) laser. The responses of this TLPFG to external refractive index, temperature, torsion, and strain were systematically investigated to determine its sensing characteristics. The external refractive index (RI) was measured to be −602.86 nm/RIU at an RI of ~1.432. The TLPFG was used to accurately measure temperatures up to 450 °C with a sensitivity of 103.8 pm/°C. The torsion and strain sensitivity of the device were 48.94 nm/(rad/mm) and −0.63 pm/µε, respectively. These results demonstrate that the proposed TLPFG could be used as sensors in a series of application fields including high temperatures and external environments.

## 1. Introduction

Optical fiber sensors have been widely used in a variety of fields due to several inherent advantages. They are highly sensitive, lightweight, compact, and immune to electric interference. Long period fiber gratings (LPFGs) are a promising passive optical fiber device, capable of coupling light in fiber core modes to several forward-propagating cladding modes, forming a series of attenuation bands in the transmission spectrum [1]. As reported in previous studies, LPFGs-based sensors have been developed to measure strain, pressure, and temperature [2]. Various fabrication methods have been used to inscribe LPFGs for specific sensing applications, including UV-light exposure [3,4], CO_2_ laser irradiation [5,6], mechanical pressure [7,8,9], electric arc discharge [10,11,12,13], femtosecond laser (Fs) [14,15].

Among these, UV-light exposure is the most common. This process takes advantage of the photosensitivity of Ge-doped glass in the fiber core to induce periodic index modulation. However, LPFGs fabricated in this way are not resistant to high temperatures and exhibit serious grating degradation, even below 100 °C [14]. In addition, pretreated high-pressure hydrogen loading processes are required to enhance the grating writing efficiency. To solve this problem, Kondo et al. firstly fabricated an LPFG in a single mode fiber (SMF) using an 800 nm Fs laser and the point-by-point method without hydrogen loading [14]. In 2008, Allsop et al. successfully inscribed a series of symmetric and asymmetric LPFGs in photonic crystal fibers (PCFs) by a low-repetition femtosecond laser system with the point-by-point method [16]. However, this point-by-point method required precise alignment of the focused laser spot and fiber core when inscribing LPFGs. Further improvements have been made to this fabrication techniques in the past decade. In 2010, Liu et al. presented a new method for fabricating LPFGs by periodically drilling holes into the photonic crystal fiber cladding using a focused infrared Fs laser [15]. Recently, Dong et al. proposed a transversal-scanning inscription method to fabricate LPFGs using an 800 nm infrared Fs laser [17]. It was determined that LPFGs fabricated with this method exhibit opposite bending characteristics compared with LPFGs inscribed with the point-by-point method.

The tilted long period fiber gratings (TLPFGs) characterized with a <90° angle between the optical axis and grating plane have attracted significant attention in the past decade [18]. Detailed theoretical analysis has shown that TLPFGs exhibit unique properties, such as the core layer guided-modes capable of coupling with a series of reverse high-order cladding modes, which did not occur in non-tilted LPFGs. Fabrication of TLPFGs can be accomplished using several different techniques. In 2011, Wu et al. experimentally fabricated TLPFGs with varying tilt angles using a CO_2_ laser [19]. The resulting grating transmission spectral shape was complex and included several smaller peaks. This can be attributed to regularity of simultaneous refractive index modulation in the fiber core and cladding. Furthermore, due to the large spot size of the CO_2_ laser, tilting effects in the grating were not obvious, leading to poor repeatability in the grating fabrication process. In 2012, Zhang et al. reported mechanical pressure-induced TLPFGs in a solid core PCF [20]. However, TLPFGs fabricated with this method often experience aging stability problems. In 2013, Chiavaioli et al. used the point-by-point method to produce specially-designed TLPFGs [21]. However, the fabrication process required a manual rotation stage for inscribing grating planes at desired angles, which limited reproducibility and efficiency.

In this study, we report on a tilted long period fiber grating (TLPFG) fabricated using an infrared Fs laser and a line-by-line inscription method. This technique has several advantages, including fabrication flexibility, high machining efficiency, and repeatability. The resulting TLPFGs exhibit a high-quality transmission spectrum compared with those written by a CO_2_ laser or mechanical pressure. A series of experiments were conducted to investigate the sensing performance of the proposed TLPFGs, which achieved a sensitivity of −602.86 nm/RIU as the external refractive index was increased from 1.402 to 1.432. The device achieved a sensitivity of 103.8 pm/°C when the temperature increased from room temperature to a high of 450 °C. The inscribed attenuation bands were also found to be sensitive to torsion and strain. The measured twisting and strain sensitivities were 48.94 nm/(rad/mm) and −0.63 pm/µε, respectively.

## 2. Tilted Long Period Fiber Grating Fabrications

**Figure 1:**
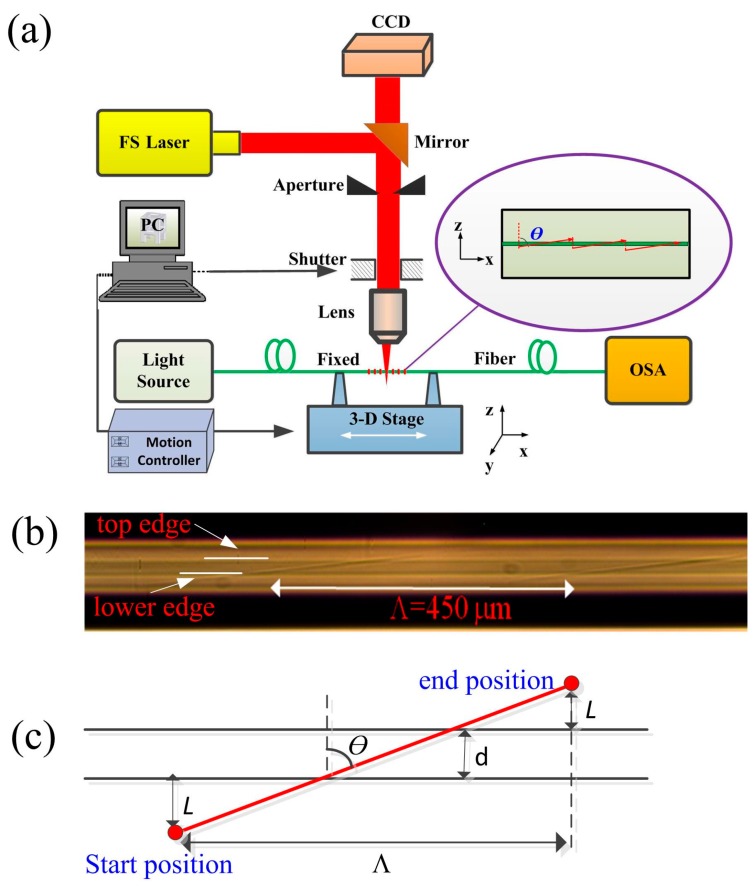
(**a**) A schematic diagram of the Fs laser micromachining apparatus; (**b**) An image of the TLPFG with a grating pitch of 450 μm; (**c**) A schematic of the Fs laser scanning trajectory.

A schematic diagram of the Fs laser micromachining apparatus is shown in Figure 1a. There are multiple primary components incorporated in the fabrication system. An Fs laser (Spectra-Physics) with a central wavelength of 800 nm, a pulse duration of 120 Fs, and a repetition rate of 1 kHz was used to provide continuous laser machining. Optical power levels were adjusted though an attenuator, which was composed of a half-wave plate and a polarizer, as discussed in Li et al. [22]. The controllable power range varied from 0 to 4 mJ. An electronic shutter (THORLABS, SC10, Shanghai, China) was connected to a computer and used to control the light path (on/off) in real time during fabrication. The microscope objective lens used in our experiments exhibited a 20× magnification and an NA value of 0.25. A 3D ultra-high precise (minimum incremental motion of 10 nm and a bi-directional repeatability of 80 nm) electric controllable translation stage (Newport XMS50, VP-25X and GTS30V, MICRO-CONTROLE Spectra-Physics S.A.S, Évry, Fracne) was used to control axial motion in the X, Y and Z directions. A charge couple device (CCD) camera was used to monitor trace movements of the laser spot and capture the morphology of the TLPFGs.

A conventional SMF (corning) was used to fabricate the TLPFGs in these experiments. During the fabrication process, a section of SMF coating layer was stripped off using fiber optic strippers and cleaned with ethanol. It was then fixed in place using fiber holders. One end of the SMF was connected to a broadband light source (BBS) and the other was directly attached to an optical spectrum analyzer (OSA) to monitor the evolution of the TLPFGs transmission spectra. The laser beam was focused in the x-y plane of the fiber core with the assistance of an optical microscope (Leica DM2500, Leica Microsystems Inc., Buffalo Grove, IL, USA) and the Z translation stage. As a result, the focused laser power could effectively alter the refractive index of the fiber core without significantly affecting the cladding. Here, we define the parameter *θ*, which represents the angle between the movement direction of the laser spot (i.e., the tilted grating plane) and the negative Y axis. The magnitude of this angle can be calculated using the following method. Firstly, we let the laser spot be at the starting point, and the vertical distance from the lower edge of the fiber core is *l*. Then using soft programming, the laser spot can scan through the fiber core from the starting position to the end position with a velocity of 50 µm/s. The vertical distance from the end position to the top edge of fiber core is also equal to *l*. The axis movement distance of the laser spot is denoted as Λ, i.e., the grating pitch. As shown in Figure 1c, we can obtain a geometric equation tan *θ* = Λ/((2*l* + d), where d is the fiber diameter of ~10 µm. By setting scanning parameters *l* = 10 µm, Λ = 450 µm, we can obtain a value of *θ* = arctan(450/(2 × 10 + 10)) = arctan(15) ≈ 86.18°. Typical laser power was set to 2.6 mW before entering the microscope objective lens. The electronic shutter was closed to obstruct the light path as the laser spot moved to the lower edge of the fiber core, to begin a new scanning cycle. This process was repeated for 30 periods, constituting a single cycle. Figure 1b shows a microscopic image taken from the top of a section of the TLPFG.

**Figure 2:**
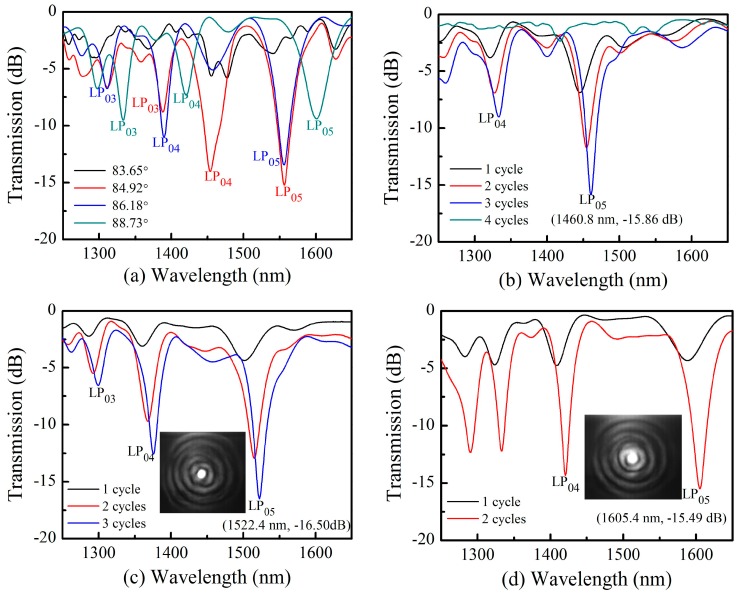
Various TLPFG transmission spectra, including (**a**) TLPFGs with varying tilted angles; (**b**) A TLPFG with a period of 420 µm; (**c**) a TLPFG with a period of 440 µm; (**d**) A TLPFG with a period of 460 µm.

This method was utilized to fabricate a series of TLPFGs with varying angles of 83.65°, 84.92°, 86.18°, and 88.73°, as shown in Figure 2a. All the TLPFGs were fabricated with the same period of 450 µm. The tilt angle was closely related to grating spectrum quality. During fabrication, the grating spectrum was nearly eliminated for the tilt angles below 83.65°. The dip in the resonant wavelength decreased noticeably for tilt angles above 88.73°. As such, there exists an optimal tilt angle for grating inscription. Yin et al. determined the optimal angle for TLPFGs formation to be ~87° [23], which is agrees well with our experimental results. In this study, a tilt angle of 86.18° as the optimum was used for fabricating three different TLPFGs with periods of 420 µm, 440 µm, and 460 µm, as illustrated in Figure 2b, Figure 2c, and Figure 2d, respectively. Larger grating periods resulted in longer resonant wavelengths under the same laser power machining conditions. This implies the resonance wavelength exhibits a red shift an increasing grating pitch. As seen in Figure 2b, the resonance wavelength dip reached a maximum value of −15.86 dB at 1460.8 nm after three scanning cycles. However, the transmission spectrum dramatically deteriorated after the fourth scanning cycle. This indicates that an over-coupling phenomenon occurred, similar to that of non-tilted LPFGs reported in ref. [24,25]. As shown in Figure 2c, the dip in the resonant wavelength around 1522.4 nm can reach up to −16.50 dB, which is a little smaller than TLPFGs fabricated by CO_2_ laser irradiation [19]. This is because the small size (~3 µm in diameter) of the focused femtosecond laser spot leads to relatively small refractive index modulation in the fiber core. Furthermore, Figure 2d demonstrates that our proposed method had a higher writing efficiency (less than 5 min), as high-quality TLPFGs transmission spectra were achieved with only two scanning cycles by optimizing writing parameters (laser power and scanning velocity). In order to better decide the cladding modes of the resonance wavelengths, we employed a mode field observation system similar to ref. [1] to observe the resonance wavelengths of 1552.4 nm and 1605.4 nm, as shown in the inset of Figure 2c and d respectively.

**Figure 3:**
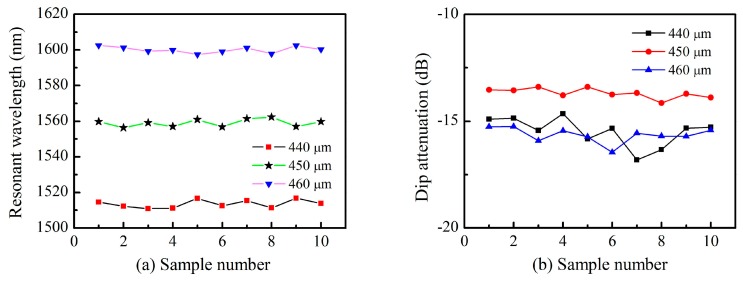
(**a**) The resonance wavelength; (**b**) The resonance depth of TLPFGs with different grating pitches.

A series of TLPFGs samples were fabricated with various grating pitches to demonstrate the repeatability of our proposed method, as shown in Figure 3. From Figure 3a we observe that variations in the resonant wavelength were within ±2 nm, which demonstrates that the refractive index modulation was relatively uniform for each sample. The depth of the resonant dip was controllable within a fluctuation range of only ±0.5 dB, as shown in Figure 3b. These results indicate that our proposed method has excellent repeatability and high stability during TLPFGs fabrication.

## 3. Refractive Index Sensing Characteristic

A series of experiments were conducted to access TLPFGs optical properties. Excluding a high temperature experiment, all tests were performed at room temperature. An ASE (Fiber Lake) light source with a wavelength ranging from 1250–1650 nm and an OSA (YOKOGAWA AQ6370C) with a minimum resolution of 0.02 nm were used in the experiments. The results provide an assessment of the sensing applications of the proposed TLPFGs design.

Conventional LPFGs are sensitive to variations in refractive index. This is caused by the formation of resonance loss peaks through coupling between the core mode and several high-order cladding modes, which are highly sensitive to outer cladding mediums. As such, TLPFGs have been used as refractive index sensors in a variety of studies [26,27,28].

In this study, refractive index response measurements were conducted using a TLPFG with a period of 460 µm and a total of 30 periods. The grating had three distinct resonance wavelengths attributed to different high order cladding modes, coupled to the core mode in a wavelength range of 1250–1650 nm. One end of the TLPFG was fixed in a fiber clamp and the other end was attached to a 25 g small weight, providing a constant strain to align the fiber along the fiber axis direction. The grating area was completely immersed in a series of commercial refractive index matching liquids (Cargille Lab—http://www.cargille.com) ranging from 1.402 to 1.442. Each grating variation spectrum profile was recorded as it was in a stable state. About 5 min are required during this process.

**Figure 4:**
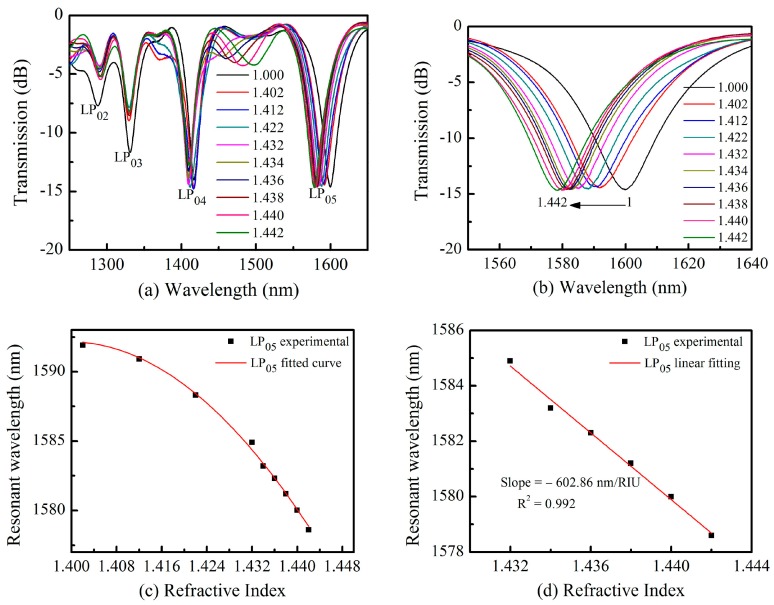
(**a**) The transmission spectrum shift of TLPFG versus the refractive index variation; (**b**) The magnified wavelength shift spectrum at 1599.7 nm; (**c**) Polynomial term fitting of resonance wavelengths as the refractive index increased from 1.400 to 1.442; (**d**) The Linear Fitting of resonance wavelength as the refractive index increased from 1.432 to 1.442.

The grating area was cleaned with ethanol after each measurement, shifting the spectrum back to its original values in air. Three resonance wavelengths were observed which exhibited different refractive index responses, as shown in Figure 4a. High-order coupled cladding modes exhibited relatively larger sensitivities. The resonance wavelength at 1559.7 nm with pattern of LP_05_ shifted ~21.08 nm toward the short wavelength regime as the refractive index increased from 1.000 to 1.442. However, the low-order resonance wavelength at 1331.24 nm with a pattern of LP_03_ only shifted ~1.24 nm. Figure 4b shows a magnified wavelength shift spectrum at 1599.7 nm. From this we observe the resonance wavelength includes an obvious shift while the transmission power showed almost no change. The relationship between refractive index and wavelength is shown in Figure 4c. The resonance wavelength follows a nonlinear relationship for refractive index between 1.000 and 1.442 and a linear relationship between 1.432 and 1.442. The resulting sensitivity of LP_05_ reached −602.86 nm/RIU with a fitting degree of R^2^ = 0.997 as shown in Figure 4d. This result is on the same order with the ultra-long period gratings inscribed using an 800 nm Fs lasers [29]. Our proposed TLPFGs exhibits a high refractive index sensitivity, compared with D-shaped fiber grating refractive index sensor induced by an 800 nm Fs laser with a sensitivity of only ~30 nm/RIU [30].

## 4. High-Temperature Sensing Characteristic

Optical fiber sensors have played an important role in the fields of defense, aerospace, and modern industry. Several studies have investigated the high-temperature properties of LPFGs inscribed in different types of optical fibers [29,31,32]. However, the high temperature response of TLPFGs has not been studied as extensively. In this section, we performed high-temperature experiments to characterize the thermal response of TLPFGs inscribed in an SMF using an Fs laser. The TLPFGs used in these experiments had a grating pitch of 440 µm and a total of 30 periods.

The TLPFG was placed in a temperature-controlled thermoelectric oven within an accuracy of ±1 °C, to observe thermally-induced variations in its grating spectral profile. During these measurements, the temperature was increased from room temperature (~25 °C) to 450 °C with a step size of 25 °C. The temperature was maintained for 10 min during each measurement to record the stabilized grating transmission spectrum using an OSA.

**Figure 5:**
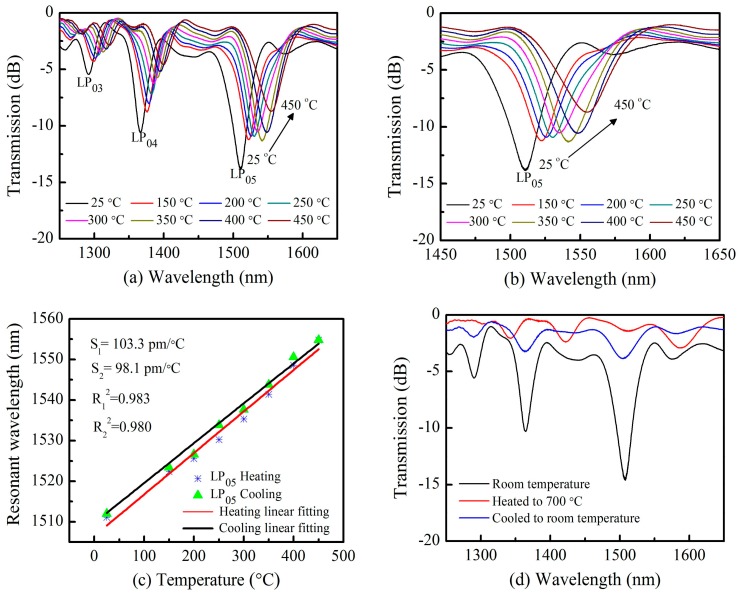
(**a**) The transmission spectrum shift of the TLPFG versus temperature variations from 25 °C to 450 °C; (**b**) The resonance wavelengths shift shifts versus temperature variations from 25 °C to 450 °C; (**c**) Polynomial term fitting of resonance wavelength as the temperature increased to 450 °C and then cooled to 25 °C again; (**d**) The transmission spectrum of a TLPFG under various temperature conditions.

As shown in Figure 5a, all three resonance wavelengths in the range from 1250 to 1650 nm shifted towards longer wavelengths as the temperature increased from 25 °C to 450 °C. It is evident that the resonance loss peak of the lower-order cladding modes (LP_04_) at 1376.3 nm decreased much faster than the high-order cladding modes (LP_05_) at 1511.9 nm. Lower-order resonance loss peaks exhibited wavelength shifts of 23.2 nm while high-order resonance loss peaks shifted ~32.8 nm over the same temperature range, as illustrated in Figure 5b. This indicates that high-order cladding resonance wavelength with pattern of LP_05_ shows a relatively high temperature response sensitivity, as high as S_1_ = 103.3 pm/°C, with good linearity (R_1_^2^ = 0.983) as shown in Figure 5c. This temperature response sensitivity is larger than many other high temperature sensors based on optical fibers [14,32]. Staying at 450 °C about 20 min, then the temperature was gradually cooled down to room temperature with a step of 25 °C. At each point, the temperature was maintained for 10 min to record the data. Linear fitting of the experimental results was conducted, achieving a fitting degree of 0.980. The slope of this fit corresponds a sensitivity of S_2_ = 98.1 pm/°C as shown in Figure 5c.

Temperature endurance was further investigated by heating the TLPFG to 700 °C and then cooling it to room temperature. Figure 5d shows variations in the transmission spectrum during this process. The grating degraded dramatically and the resonance dip disappeared entirely as the temperature approached 700 °C, indicating the TLPFG could no longer function as an optical waveguide. The reason may be that the Fs laser-induced index change here relaxes above 700 °C as reported in reference [14]. As the TLPFG was cooled down to room temperature, its transmission spectrum was gradually restored but the resonance dip remained in a deteriorated state, due to the effects of high temperature. It also indicates that the high temperature (>500 °C) may degrade the gratings spectra [14]. The proposed TLPFG maintained a high-quality transmission spectrum demonstrating excellent thermal stability as the temperatures below 450 °C. In contrast, TLPFGs fabricated with UV-light exhibit grating degradation at temperatures below 100 °C. The primary reason is that refractive index changes caused by the focused irradiation of femtosecond pulses differ from those induced by UV-light induced [14]. As such, TLPFGs inscribed by Fs laser could be used for potential applications in the field of high temperature sensing.

## 5. Torsion Sensing Characteristic

Optical fiber torsion sensors have been widely used in the automotive industry and for anthropomorphic robotics applications, due to their advantages of being lightweight and immune to electronic interference [33]. Several optical-fiber-based torsion sensors have been reported in the past few years, including corrugated LPFGs, UV radiation-induced tilted fiber gratings, and Sagnac interferometers [34]. However, many of these devices cannot effectively determine the direction (clockwise or counter-clockwise) of the applied torsion, which limits their practical application. For this reason, high-sensitivity sensors have been investigated to distinguish both the torsion angle and direction simultaneously.

**Figure 6:**
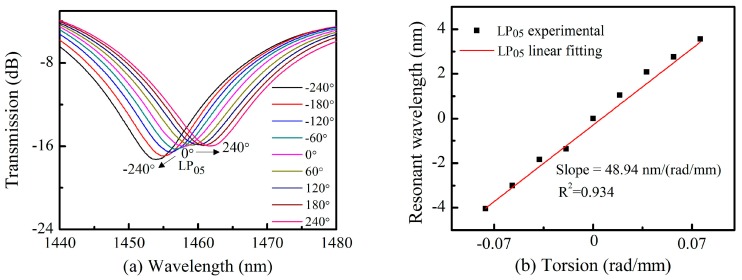
(**a**) Resonance wavelength shifts for TLPFGs under an applied torsion in the range of −0.076 rad/mm to +0.076 rad/mm; (**b**) A linear fitting between the torsion and wavelength shift.

An experimental setup similar to that of Deng et al. [33] was used to investigate the torsion sensing properties of our proposed TLPFGs. Measurements were conducted using electronic controllable rotators which could twist the grating samples in two different rotate directions with a minimum angle of 0.1°. The distance between these two rotators (the twist length L) was equal to 55 mm in our experiments. The rotation angle was denoted as α. Thus, the applied torsion τ could be written as τ = α/L = α/0.055 ≈ 18α (rad/m). The rotation angle α was measured from 0° ± 240° with an interval of 60°. Positive angles represented the clockwise rotation, while negative angles indicated counter-clockwise rotation. Figure 6a shows resonance wavelength shifts in the transmission spectra as the torsion τ varied from −0.076 rad/mm (−240°) to +0.076 rad/mm (+240°). After each test, the spectrum was shifted back to its original values (0°). This process will cost about 2 min. It is evident that the resonance wavelength with pattern of LP_05_ (as shown in Figure 2b shows a red shift when the applied torsion is clockwise, and vice versa. Wavelength shifts are plotted in Figure 6b. An approximately linear relationship (R^2^ = 0.934) was observed between the wavelength shift and the applied torsion during both clockwise and counterclockwise rotation. As such, a linear fitting was applied to the data. The resulting slope quantifies TLPFG torsion sensitivity, reaching a value of 48.94 nm/(rad/mm). This is twice as high as conventional non-tilted LPFGs inscribed in an SMF, with an average of ~23 nm/(rad/mm) [33].

## 6. Strain Sensing Characteristic

Applying strain to LPFGs along the axial direction leads to axially stretching, which causes variations in the grating pitch Λ and the refractive index of both the core and cladding, due to photo-elastic effects in the fiber. This induces resonant wavelength shifts in LPFGs, which have been used as strain sensors in multiple studies [35]. However, the strain properties of TLPFGs have not been investigated as thoroughly. In this experiment, the employed TLPFG sample had a grating pith of 440 µm and a total of 30 periods. It posed LP_05_ cladding modes around the resonance dip of 1516.8 nm similar to Figure 2c.

**Figure 7:**
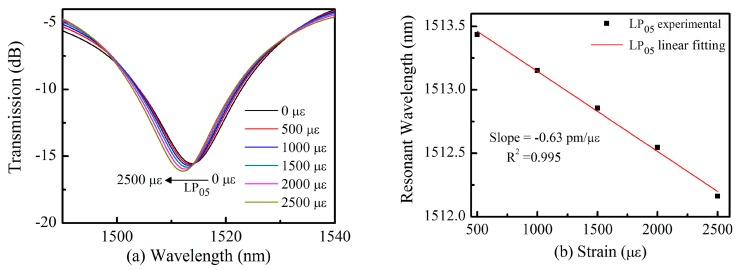
(**a**) TLPFG resonance wavelength shifts under an applied strain ranging from 0 to 2500 με; (**b**) A linear fitting between the strain and wavelength shift.

The strain sensitivity of the proposed TLPFG was investigated, using the strain measurement setup discussed in our previous publication [36]. Two ends of the grating area were fixed by a two- micrometer translation stage with a 5 µm resolution. The distance between them was 10 cm, and the minimum strain change was Δε = 100 µε. As shown in Figure 7a, the resonance wavelength with pattern of LP_05_ exhibited a blue shift for the applied strains ranging from 0 to 2500 µε, as recorded by an OSA with a resolution of 0.02 nm. This implies that TLPFG can withstand a strain of 2500 µε without breaking. It is noted that at each test point we need about 2 min to record the stable transmission spectrum. Linear fitting of the experimental results was conducted, achieving a fitting degree of 0.995 as shown in Figure 7b. The slope of this fit corresponds a strain response of −0.63 pm/µε, which is higher than the sensitivity of LPFG etched in SMFs by CO_2_ laser pulses (−0.45 pm/με [37]). This result support the use of TLPFGs as a strain sensor in practical applications.

## 7. Conclusions

We have demonstrated a TLPFG fabrication method using an infrared femtosecond laser and a line-by-line inscription technique. The resulting TLPFG exhibited a high-quality transmission spectrum and excellent repeatability. The external refractive index, temperature, twist, and strain sensing characteristics of the TLPFG were investigated experimentally. As the surrounding refractive index gradually increased, the resonance wavelength of the TLPFG exhibits a blue shift with a sensitivity of −602.86 nm/RIU at an external RI of ~1.435. The TLPFG could withstand a high temperature of up to 450 °C, with a temperature sensitivity of 103.8 pm/°C. It could also be used as a torsion sensor capable of determining the twisting direction. Consequently, it is anticipated that the TLPFG-based sensors could be used for a series of practical engineering applications.
